# First Report on the Artificial Cultivation Techniques of *Buchwaldoboletus xylophilus* (Boletales, Boletaceae, *Buchwaldoboletus*) in Southwest China

**DOI:** 10.3390/jof11030172

**Published:** 2025-02-21

**Authors:** Tianwei Yang, Hongjun Mu, Liming Dai, Jing Liu, Xinjing Xu, Feng Gao, Yiwei Fang, Sipeng Jian, Mingxia He, Chunxia Zhang

**Affiliations:** Yunnan Institute of Tropical Crops, Jinghong 666100, China; yangtianweizj@126.com (T.Y.); mhjyitc@139.com (H.M.); limingdai@126.com (L.D.); ljxsbn@126.com (J.L.); 15198689253@163.com (X.X.); gaofeng19860704@outlook.com (F.G.); fangyiwei11@163.com (Y.F.); a1464993369@gmail.com (S.J.); hemingxia@yitc.com.cn (M.H.)

**Keywords:** artificial cultivation, boletes, *Buchwaldoboletus xylophilus*, fruiting body, edible mushrooms

## Abstract

*Buchwaldoboletus xylophilus* is an edible bolete species belonging to the family Boletaceae and the genus *Buchwaldoboletus*. It is found in tropical and subtropical regions, which are known for their rare wild resources. In this study, wild *B. xylophilus* was isolated and cultured, and its biological characteristics and artificial cultivation techniques were studied. The results show that the optimal carbon source, nitrogen source, and inorganic salt for the mycelium growth of *B. xylophilus* were maltose, ammonium tartrate, and magnesium sulfate, respectively. The most appropriate temperature was 28 °C, and the pH value was between 5 and 6. The most effective combination was determined via orthogonal experimentation, as follows: dextrose, ammonium nitrate, potassium dihydrogen phosphate, and 28 °C. The results of artificial cultivation in mushroom houses show that the mycelium of *B. xylophilus* was strong and grew well on the culture medium. The mycelial growth rate was 4.54 mm/d, and the fungus bags were filled about 50 days after inoculation. The primordia formed 9 to 14 days after covering with soil and the fruiting body matured in 6~8 days. The average yield of fresh mushrooms reached 131.07 ± 29.38 g/bag, and the average biological efficiency reached 28.48 ± 6.39%. In this study, artificial cultivation technology in respect of *B. xylophilus* in mushroom houses is reported for the first time. The fruiting bodies obtained through cultivation were identified using morphological and molecular biological methods. This technology offers benefits such as affordability, a brief cultivation cycle, substantial yields, and superior quality, making it ideal for industrial-scale and extensive cultivation.

## 1. Introduction

Bolete species comprise an important group of macrofungi [1,2,3]. China has abundant bolete resources, and nearly 500 species have been reported [4,5,6]. The majority of bolete species are mycorrhizal fungi, which can establish a mutually beneficial symbiotic relationship with various higher plants, including Pinaceae, Salicaceae, Betulaceae, Casuarinaceae, Fabaceae, and Dipterocarpaceae [7,8,9,10,11]. Therefore, the artificial cultivation of boletes remains challenging. At present, only a few types of boletes, such as *Phlebopus portentosus* and *Phlebopus spongiosus*, can be artificially cultivated [12,13]. In 1969, Pilat took *Buchwaldoboletus lignicola* as a type species and formally established the genus Buchwaldoboletus, classifying it into the Boletaceae family [14]. This genus is distributed all over the world, and 12 species have been described so far [15]. Predominantly, *Buchwaldoboletus* species are wood-rot fungi, typically found growing on decomposed wooden stakes, decomposed rubber sawdust, and in bamboo forests [16,17,18,19].

*Buchwaldoboletus xylophilus* (Petch) Both & B. Ortiz is an infrequently encountered bolete species native to tropical regions. It has a wild fruiting body and is exceptionally rare; it is known to occur in select locations within China, such as Xishuangbanna, Hainan, and Hong Kong [15,19]. It is also distributed in Sri Lanka, Malaysia, India, and the Philippines [15,20,21,22]. In 2020, we collected a rare species of bolete in the rubber woodland of Xishuangbanna Dai Autonomous Prefecture, Yunnan Province. Through morphological and molecular biological identification, it was determined that the species is a member of the family Boletaceae and the genus *Buchwaldoboletus*, named *B. xylophilus* (Petch) Both & B. Ortiz [19]. *B. xylophilus* is an edible mushroom with a delicious taste and a crispy texture. In the Yunnan region, it can be sold at a price ranging from CNY 60 to 100 per kilogram (approximately USD 8 to 14). Additionally, the market demand for it is expected to continuously expand.

The fruiting body of *B. xylophilus* is of moderate size, can be found either solitarily or in clusters, and boasts an aesthetically pleasing mushroom form. This species also displays a cap that is yellow-brown to brown and a stipe that ranges from yellow-brown to reddish-brown. This species also displays a distinctive yellow mycelium at the stipe’s base. Additionally, its spores are diminutive, and typically oval or spherical in shape [19]. The flesh is yellowish and turns blue rapidly after injury. As time passes, the color of the injured part gradually darkens [19].

*B. xylophilus* is an endangered species with few wild resources and a low wild yield. Therefore, the biological characteristics and cultivation of *B. xylophilus* were studied, laying a foundation for the resource protection as well as development and utilization of the species.

## 2. Materials and Methods

### 2.1. Isolation

**Figure 1:**
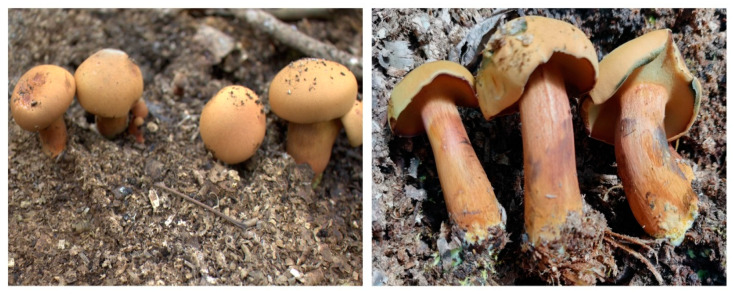
Fruiting bodies of wild *B. xylophilus.*

Fruiting bodies of wild *B. xylophilus* were collected from the rubber forest of Jinghong City, Xishuangbanna Dai Autonomous Prefecture, Yunnan Province (Figure 1). The morphological structure was recorded. The soil particles at the root of the fruiting body were removed. Mycelia were isolated from the fruiting bodies and cultured on PDA medium (potato 200.0 g, dextrose 20.0 g, yeast extract 2.0 g, MgSO_4_ 1.0 g, KH_2_PO_4_ 1.0 g, agar 18.0 g, water up to 1 L, pH not adjusted). The isolated strains were named YITC-BU001, YITC-BU002, and YITC-BU003. The YITC-BU001 strain was adopted for the experiments on biological characteristics. Mycelial cultures were incubated at 28 °C for 15 days. The cultures were kept on PDA slants at 15 °C.

### 2.2. Effect of Carbon and Nitrogen Sources

To assess mycelial growth, six different carbon source media were utilized. PDA medium (without dextrose) was added with a 20 g/L carbon source comprising one of the following: dextrose, fructose, sucrose, mannitol, maltose, or soluble starch. The medium was sterilized by autoclaving at 121 °C for 30 min. A total of 20 mL of the medium was dispensed into each 9 cm diameter Petri dish. A 5 mm diameter plug of mycelium from the edge of a 15-day-old culture was inoculated on the medium. Each Petri dish was sealed with Parafilm to mitigate contamination risks. Incubation of the cultures was carried out in the dark at 28 °C. There were 10 replicates for each treatment, and the colony diameter was measured every 5 days.

Six distinct nitrogen source media were employed to evaluate mycelial growth. PDA medium (without yeast extract) was added with a 2 g/L nitrogen source comprising one of the following: tryptone, beef extract, yeast extract, ammonium tartrate, KNO_3_, or NH_4_NO_3_. The subsequent procedures were identical to those of the carbon source experiment.

### 2.3. Effect of Inorganic Salt

Six inorganic salt media were used to test the growth ability of mycelia. PDA medium (without MgSO_4,_ KH_2_PO_4_) was added with 1 g/L inorganic salt comprising one of the following: NaCl, MgSO_4_, KH_2_PO_4_, MnSO_4_, CaSO_4_, or NaNO_3_. Cultures were incubated in darkness at 28 °C. There were 10 replicates for each treatment, and the colony diameter was measured every 5 days.

### 2.4. Effect of Temperature and pH

Plugs of mycelium 5 mm in diameter from the edge of a 15-day-old culture were inoculated on PDA medium plates and then incubated at 15, 20, 23, 25, 28, 30, or 35 °C in darkness. There were 10 replicates for each treatment, and the colony diameter was measured every 5 days.

Plugs of mycelium 5 mm in diameter from the edge of a 15-day-old culture were inoculated on PDA medium plates, and different pH values were established. The pH was adjusted to 4.0, 5.0, 6.0, 7.0, or 8.0 with 0.1 mol/L NaOH or 0.1 mol/L HCL. The cultures were incubated in darkness at 28 °C. There were 10 replicates for each treatment, and the colony diameter was measured every 5 days.

### 2.5. Orthogonal Experiment

According to the screening results for the carbon source, nitrogen source, inorganic salt, and temperature conditions, the best three levels were selected for each condition, and an orthogonal experiment with four factors and three levels was carried out.

### 2.6. Preparation of Liquid Culture

Amounts of 200 g potato and 20 g bran were boiled with 1 L of water for 20 min, and the filtrate was collected. Amounts of 15 g dextrose, 2 g yeast extract, 1 g MgSO_4_, 1 g KH_2_PO_4_, and 2 tablets of vitamin B_1_ (including 10 mg of Vitamin B_1_) were added to the filtrate and dissolved; pH was not adjusted. The liquid medium was added to 1 L and sub-packed into a 500 mL triangular flask containing 250 mL of liquid medium. The medium was autoclaved at 121 °C for 30 min. Five 0.5 cm^3^ plugs of mycelium were inoculated in the liquid medium. Cultures were incubated on a shaker (140 rpm/min) in darkness at 28 °C for 7 days.

### 2.7. Solid Substrate Inoculums

The cultivation medium contained 40% rubber sawdust, 35% rubber wood bits, 15% bagasse, 8% corn flour, 1% sucrose, and 1% calcium carbonate. The cultivation medium components were mixed in their respective proportions, and water was added to moisten the medium, adjusting the water content to 60~65%. Each polypropylene bag was filled with 1.0 kg of the cultivation medium. The medium was autoclaved at 121 °C for 2 h and cooled to ambient temperature before inoculation. A volume of 10 mL of liquid inoculum was inoculated into each cultivation bag (17 × 35 × 0.07 cm). The substrate was cultured in darkness at 28 °C. The mycelium grew from the inoculation surface as the starting point and grew downward to fill the bag as the end point. The distance from the inoculation site to the point of full colonization was measured to calculate the mycelial growth rate within the cultivation medium.

### 2.8. Fructification

The casing materials were composed of peat and vegetable garden soil mixed at a 1:1 volume ratio, with a casing layer with a thickness of about 3–4 cm. Once the bags were fully colonized by mycelium, their surfaces were cased with the soil mixture. The cased mushroom bags were transferred to the mushroom cultivation house. The temperature in the mushroom cultivation house was maintained at 28–30 °C, and the relative humidity was 80~85%.

When the mycelium had grown to the surface of the casing layer and the primordia had formed, the cultures were subjected to a temperature of 27–29 °C and a light intensity of 1000 Lx in a 12 h light/dark cycle. The quantities of primordia formed within each bag, as well as those of the mature fruiting bodies, were duly recorded. Subsequently, the yield of fresh mushrooms was measured and the biological conversion rate was calculated. Biological efficiency = Fresh mushroom yield ÷ Cultivation medium dry weight × 100%.

### 2.9. Morphological Observation and Molecular Identification

The morphological characteristics of the fruiting bodies of the artificially cultivated *B. xylophilus* were observed and described. The morphologies of mycelia and basidiospores were observed under a Zeiss microscope (ZEISS AxioScope A1, Carl Zeiss (Shanghai) Management Co., Ltd., Shanghai, China) at a magnification of 40 times (40×).

Genome DNA of the fruiting bodies of the artificially cultivated *B. xylophilus* strains YITC-BU001, YITC-BU002, and YITC-BU003 was extracted using a plant genome extraction kit (Beijing Tsingke Biotech Co., Ltd., Beijing, China). Using ITS5 (5′-GGAAGTAAAAGTCGTAACAAGG-3′)/ITS4 (5′-TCCTCCGCTTATTGATATGC-3′) and LR0R (5′-ACCCGCTGAACTTAAGC-3′)/LR5 (5′-TCCTGAGGGAAACTTCG-3′) as primer pairs, the ITS and 28S rDNA fragments of the strain were amplified via PCR. The parameters of the PCR reaction were as follows: pre-denaturation at 98 °C for 2 min; 98 °C for 10 s, 56 °C for 10 s, and 72 °C for 10 s, for a total of 35 cycles. After amplification at 72 °C, the reaction was extended for 5 min. After the PCR amplification product was detected using 1.2% agarose gel electrophoresis, the DNA was purified and recovered via a purification kit, and the amplification products were sent to Qingke Biotechnology Co., Ltd., Kunming, China for gene sequencing. The sequencing results were spliced using Contig Express (V3.0.0) software and submitted to GenBank. Based on the outcomes of the BLAST online comparison, strains featuring high coverage and similarity were chosen, and a phylogenetic tree was constructed through cluster analysis using the software MEGA 6.0.

### 2.10. Data Analysis

SPSS 20 software was used to conduct a statistical analysis on the data related to the effects of different nutritional and environmental conditions on the mycelial growth rate of *B. xylophilus*.

## 3. Results

### 3.1. Strain Isolation and Mycelial Growth

**Figure 2:**
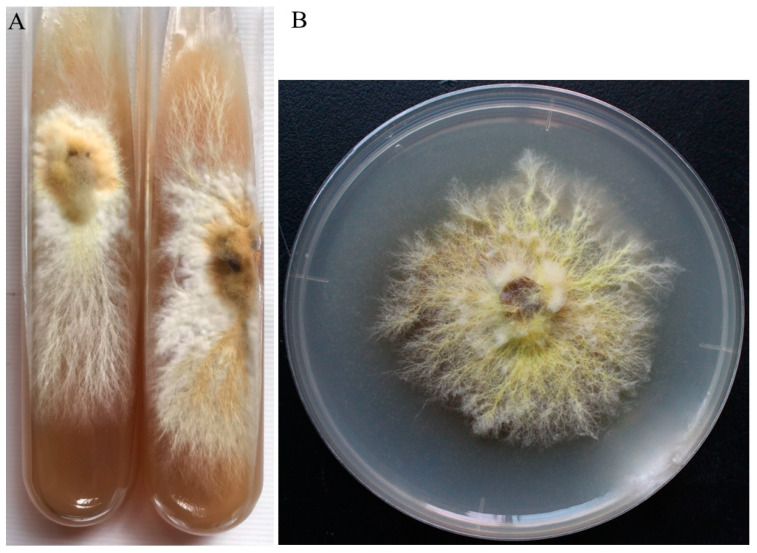
*B. xylophilus* strain. (**A**) Slant culture; (**B**) Petri dish culture.

**Figure 3:**
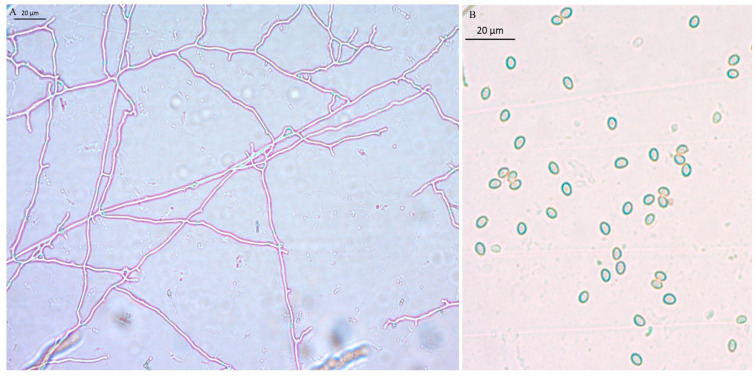
Microstructure of the mycelium and basidiospores of *B. xylophilus*. (**A**) The mycelium of *B. xylophilus* without clamp connection. (**B**) Basidiospores.

The preserved slant cultures were inoculated onto PDA Petri dishes (9.0 cm). Upon cultivation on the medium, the colonies achieved a diameter of 7.0 cm within 15 days. The periphery of the mycelium exhibited hues ranging from pale yellow to bright yellow, presenting a robust and well-defined appearance (Figure 2). The mycelium near the inoculation point was yellow and white, and fluffy. The mycelium has a faint and delicate fragrance. There were no liquid droplets or sclerotia formations during the culture process. Under microscopic examination, the mycelium of *B. xylophilus* was observed to lack a clamp connection (Figure 3A). Basidiospores were 4.5–5.5 × 3–4 μm in size and identified as being elliptical or circular in shape (Figure 3B).

### 3.2. Effect of Carbon and Nitrogen Sources on Mycelial Growth

*B. xylophilus* was observed to grow on all media. The mycelia grew fastest on the medium with maltose as the carbon source, with a growth rate of 1.70 ± 0.07 mm/d (Table 1). The mycelium was thick, dense, and yellow. The mycelium also grew well on the medium with dextrose as the carbon source, with a growth rate of 1.63 ± 0.07 mm/d. When cultivated on a medium with sucrose or soluble starch serving as the carbon source, the mycelial growth was observed to be slower, sparse, and lacking vigor, with growth rates as low as 0.64 ± 0.14 mm/d and 0.91 ± 0.09 mm/d, respectively. The selection of the carbon source is very important in the artificial cultivation of *B. xylophilus*, and maltose is the best carbon source.

Ammonium tartrate can promote the mycelial growth of *B. xylophilus* (Table 2). The most rapid mycelial growth was noted on the medium where ammonium tartrate was utilized as the nitrogen source, achieving a growth rate of 1.77 ± 0.04 mm/d. Additionally, the mycelium developed the greatest density on this medium. The mycelium thrived on the medium supplemented with ammonium nitrate as a nitrogen source, exhibiting a growth rate of 1.58 ± 0.13 mm/d. However, the edge of the mycelium grew sparsely. The mycelial growth rate was the slowest on the medium with potassium nitrate as the nitrogen source. Ammonium nitrate was the most suitable nitrogen source.

### 3.3. Effect of Inorganic Salt on Mycelial Growth

The mycelial growth was most vigorous on the medium containing MgSO_4_ as the inorganic salt, with a growth rate of 1.49 ± 0.06 mm/d (Table 3). The mycelial growth rate was the slowest on the medium with MnSO_4_ as the inorganic salt, with a growth rate of 0.95 ± 0.09 mm/d. There were no significant differences between the NaCl, KH_2_PO_4_, CaSO_4_, and NaNO_3_ inorganic salt media.

### 3.4. Effect of Temperature and pH on Mycelial Growth

The mycelium did not grow at 15 °C (Table 4). At a temperature of 20 °C, the mycelium exhibited a significantly slow growth rate of 0.49 ± 0.08 mm/d. As the temperature increased, the growth rate of the mycelium also increased. When the temperature reached 28 °C, the mycelial growth rate was the fastest, at 1.63 ± 0.10 mm/d, and the mycelium was thick and dense. A fast rate of mycelial growth was also observed at 30 °C, but the mycelium was found to be sparse with irregularly shaped edges. When the temperature reached 35 °C, the mycelium underwent fragmentation, adopted a white appearance, and exhibited irregular growth patterns. Considering the mycelial growth rate and mycelial growth vigor, the optimal temperature was 28 °C.

The pH had a certain influence on the mycelial growth of *B. xylophilus*. The mycelial growth rate was the fastest at pH values of 5 and 6, reaching 1.42 ± 0.14 mm/d and 1.41 ± 0.07 mm/d, respectively (Table 5). There was no significant disparity between the two; therefore, the optimal pH range was from 5 to 6. When the pH was 7 or above, the mycelium became weakened and turned yellow-white. *B. xylophilus* is more favorably adapted for growth in acidic environments.

### 3.5. Orthogonal Experiment

Temperature had the greatest impact on the mycelial growth of *B. xylophilus* (Table 6). The temperature range was the largest, at 0.67. The second largest was the nitrogen source, with a range of 0.31. The influence of carbon sources and inorganic salts on the mycelial growth of *B. xylophilus* was minimal. The optimal conditions for the mycelial growth of *B. xylophilus* are dextrose, NH_4_NO_3_, KH_2_PO_4_, and 28 °C.

### 3.6. Cultivation of B. xylophilus for Fruiting Bodies

**Figure 4:**
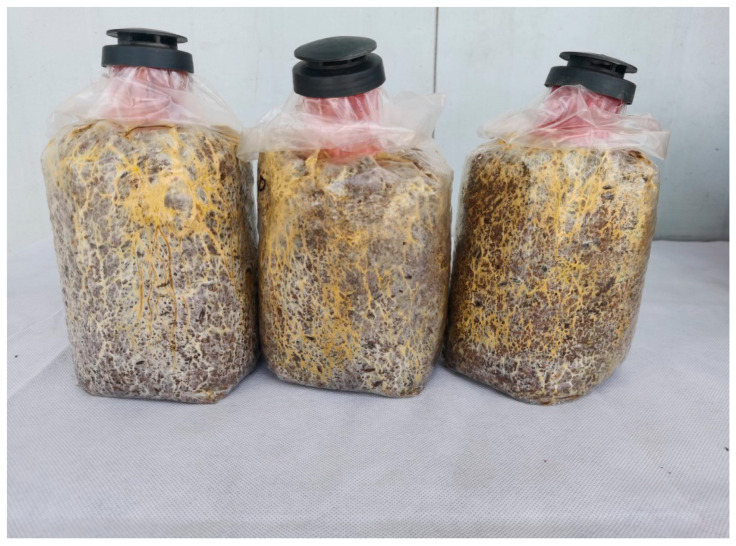
Mycelium growth of *B. xylophilus* on the cultivation substrate.

The mycelium of *B. xylophilus* was robust and appeared light yellow or yellow on the cultivation medium (Figure 4). The mycelial growth rate was 4.54 mm/d, and the mycelium filled the bags about 50 days after inoculation.

**Figure 5:**
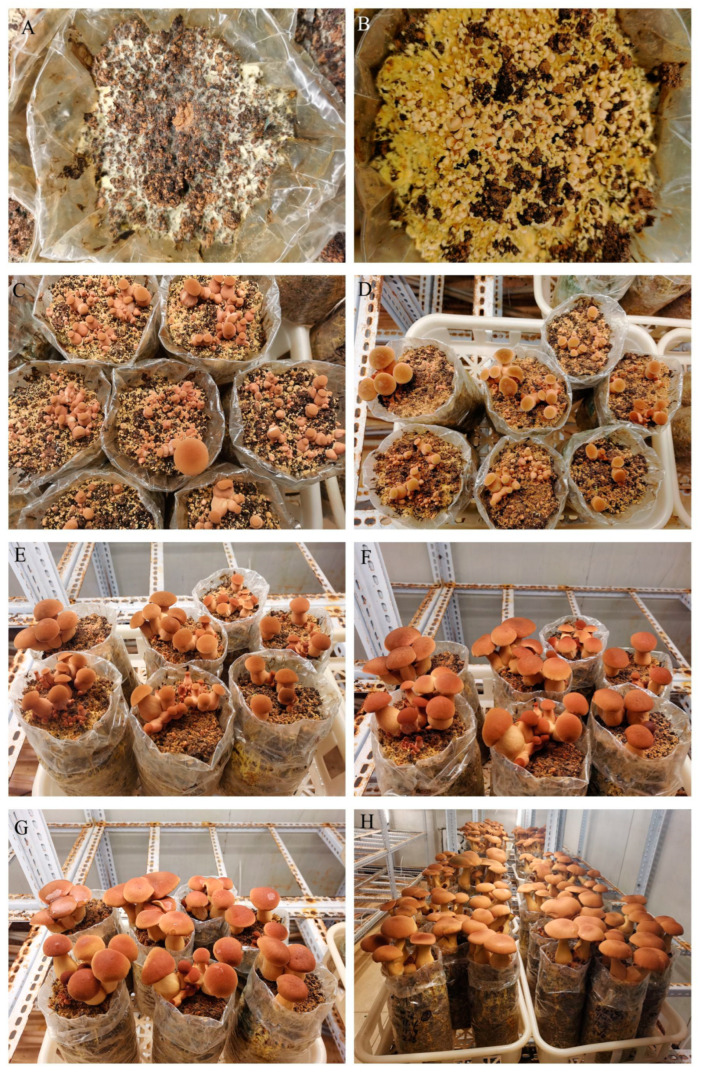
Growth process of fruiting bodies of *B. xylophilus*. (**A**) Mycelia grows to the surface of covering soil layer; (**B**) primordium; (**C**–**F**) young mushrooms; (**D**–**G**) changes in fruiting body growth at intervals of 24 h; (**G**,**H**) mature fruiting bodies.

After casing the soil for 7–12 days, the yellow mycelium grew to the surface of the soil layer (Figure 5A). The cultures were then incubated at 27~29 °C with 1000 Lx light (12 h light/12 h dark). After 2 days, primordia had formed on the surface (Figure 5B). When the primordia grew to 1–2 cm, the pileus and the stipe began to differentiate and enter the stage of young mushroom growth (Figure 5C–F). About 6–8 days after the formation of the primordia, the fruiting body matured (Figure 5G–H). When the height of the fruiting body reached 8–12 cm and the pileus had not yet flattened, the fruiting bodies could be harvested.

The average time for the formation of *B. xylophilus* primordia after covering with soil was 11.28 ± 1.50 days. A large number of primordia were densely spread on the surface of the overlying soil layer. The average numbers of young mushrooms and mature fruiting bodies per bag were 5.70 ± 3.69 and 4.13 ± 2.21, respectively. The average fresh mushroom yield of *B. xylophilus* was 131.07 ± 29.38 g/bag, and the average biological efficiency was 28.48 ± 6.39% (Table 7).

### 3.7. Identification of Cultivated Mushrooms

#### 3.7.1. Morphological Analysis

**Figure 6:**
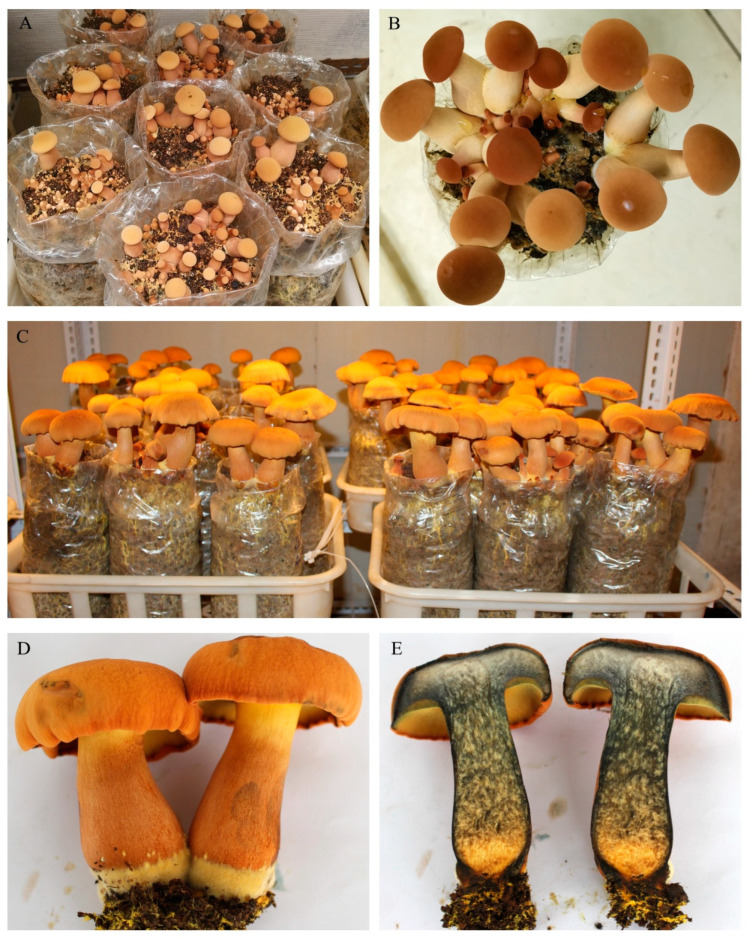
Morphology of fruiting bodies of artificially cultivated *B. xylophilus*. (**A**,**B**) Growing young mushrooms; (**C**,**D**) mature fruiting bodies; (**E**) longitudinal section of fruiting body.

Compared with the wild fruiting bodies, the cultivated fruiting bodies of *B. xylophilus* had a similar morphology, yet they possessed a more vibrant hue (Figure 6A–C). The height of the fruiting body was 6~19 cm. The pileus was orange, with a diameter of 5~13 cm. The context and tubes were yellow. The thickness of the context was 1~2.5 cm. The stipe was yellowish brown, with yellow villous hyphae at the base of the stipe (Figure 6D). The fruiting body quickly turned light blue or blue after injury (Figure 6E).

#### 3.7.2. Characteristics of Phylogeny

**Figure 7:**
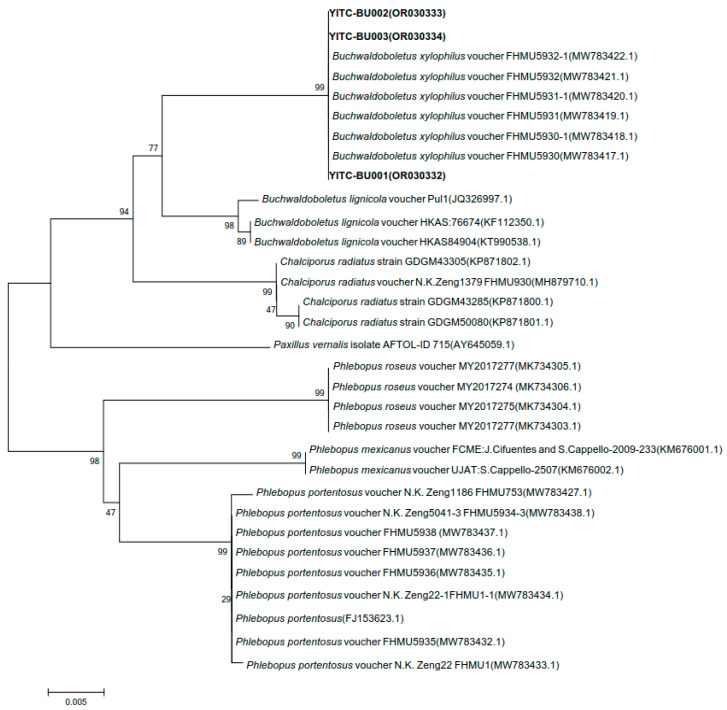
Phylogenetic analysis based on 28S gene sequence.

BLAST was used for the homology search, and a similarity comparison was performed with the sequences of near-source strains in the database (Table 8). Strain YITC-BU001’s 28S rDNA gene sequence exhibits the highest similarity, with a value of 99.77%, to the wild *B. xylophilus* voucher FHMU5931-1 from Yunnan. The 28S rDNA gene sequences of strains YITC-BU002 and YITC-BU003 have the highest similarity with the *B. xylophilus* voucher X.H. Deng1 (FHMU5848) from Hainan, with values of 99.88% and 99.76%, respectively. The 28S gene sequence of the effectively published strains with high coverage and similarity was selected for phylogenetic analysis. From the clustering results, it was observed that the cultivated strains YITC-BU001, YITC-BU002, and YITC-BU003 in this study were clustered on the same branch as the wild *B. xylophilus* reported from Hainan and Yunnan (Figure 7).

**Figure 8:**
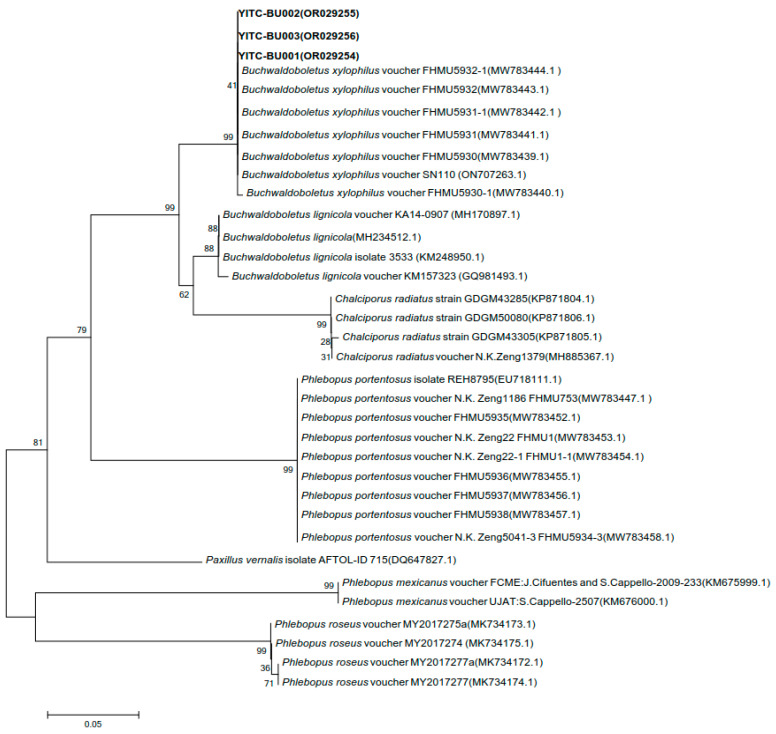
Phylogenetic tree constructed using ITS genes.

The ITS gene sequences of the strains YITC-BU001, YITC-BU003, and YITC-BU002 exhibited the highest similarities with the wild *B. xylophilus* vouchers FHMU5932 and FHMU5933-1 from Yunnan, at 99.14%, 100%, and 99.12%, respectively. The phylogenetic tree was constructed using the ITS genes of strains with high ITS similarity and effective publication. The results show that YITC-BU001, YITC-BU002, and YITC-BU003 were clustered on the same branch as the wild *B. xylophilus* reported from Hainan and Yunnan (Figure 8).

## 4. Discussion

The genus *Buchwaldoboletus* does not comprise ectomycorrhizal species like most fungi of the family Boletaceae. Instead, it is characterized as saprophytic, lignicolous, or parasitic [14,15,31]. This may be the reason why its species can grow and fruit on culture. In this study, the mycelium growth of *B. xylophilus* was influenced by different carbon sources, nitrogen sources, and inorganic salts. The optimal combination was determined through orthogonal experimentation and is as follows: dextrose, ammonium nitrate, and potassium dihydrogen phosphate. The results are consistent with the optimal carbon sources for the mycelial growth of *Phlebopus portentosus* and *Boletus edulis*, yet different in respect of nitrogen sources and inorganic salts [12,32]. Cheng et al. [33] reported that dextrose is the best carbon source for most macrofungi. *B. xylophilus* prefers ammonium nitrogen, such as ammonium tartrate and ammonium nitrate, while other edible fungi (such as *Clitocybe fragrans*, *Polyporus tuberaster*, and *Flammulina filiformis*) prefer organic nitrogen sources such as peptone, beef extract, and yeast powder [33,34,35]. This indicates that various mushroom species have distinct nutritional requirements.

In this study, the optimal growth temperature for *B. xylophilus* was determined to be 28 °C and the optimal pH was within the range 5 to 6. When the temperature was below 15 °C, the mycelium could not grow, and when the temperature was above 30 °C, the mycelium was sparse, white, and grew irregularly. This is similar to the optimal growth temperature and pH value for *P. portentosus* and *B. edulis* mycelium [12,36]. *P. spongiosus* has the highest mycelial growth rate and biomass at 30 °C and a pH value of 5 [13], while some edible mushrooms have lower mycelial growth temperatures, such as *Leccinum aurantiacum* and *Lepista sordida* [37,38].

Mycorrhizal edible fungi, such as *B. edulis*, *Tricholoma matsuake*, *Tuber indicum*, and *Thelephora ganbajun*, are highly valued for their unique flavors and potential health benefits [39,40,41,42]. These fungi form symbiotic relationships with the roots of specific host plants, a process that is essential for their growth and reproduction [43]. The complexity of this symbiotic relationship poses distinctive challenges in the field of artificial cultivation. Artificial cultivation represents a crucial approach for the preservation, advancement, and utilization of wild edible fungi. It can not only protect endangered species, but also fulfill sampling needs for scientific research. Some scholars have been trying to research the cultivation of wild bolete mushrooms [44]. However, the majority of boletes fall into the category of ectomycorrhizal fungi. They form a mycorrhizal structure in association with higher plants like those in the Pinaceae and Fagaceae families. This characteristic makes them difficult to cultivate artificially in mushroom houses [45,46,47,48].

The genus *Phlebopus* is a tropical and subtropical unique edible fungus that usually grows with *Citrus maxima*, *Mangifera indica*, *Eriobotrya japonica*, and other fruit trees in the wild [49,50,51]. *P. portentosus* and *Phlebopus roseus* can form a special insect gall structure with plant roots and scale insects [30,52,53,54]. *P. portentosus* is the first edible bolete in the world to have been cultivated industrially. At present, China has realized year-round industrial cultivation and production, with the daily output of fresh mushrooms reaching up to 16 tons [12,55]. The method for the artificial cultivation of *P. spongiosus* has been reported, and the yield has been increased through high-voltage pulsed stimulation [13,56]. *P. portentosus* is not a typical saprophytic edible fungus. It has certain saprophytic properties; however, its genome lacks or only has a small number of genes related to lignin and cellulose degradation [57,58]. Cultivation research, in respect of other bolete species, is still in the initial stage. Research into *B. edulis*, *Boletus tomentipes*, and *Boletus albidus* is in the screening and optimization stage for the mother culture medium and mycelial growth conditions, and mature fruiting bodies have not yet been cultivated in mushroom houses [59,60].

*Buchwaldoboletus* is different from *Phlebopus*, and they can be distinguished by their habitats [17]. The molecular data from this study confirm their genetic remoteness from one another. In addition, *P. portentosus* and *P. spongiosus* will form a large number of sclerotia in a wild and pure culture [13,61], while *B. xylophilus* has not been observed to form sclerotia, either in its natural environment or under artificial cultivation conditions.

Separation from host plants is a necessary condition for the industrial year-round cultivation and production of edible fungi. In this study, the artificial cultivation of *B. xylophilus* in a mushroom house was successfully achieved. The mycelium *of B. xylophilus* grows strongly in a culture medium containing 40% rubber sawdust, 35% rubber wood bits, 15% bagasse, 8% corn flour, 1% sucrose, and 1% calcium carbonate. The mycelia of *P. portentosus* and *P. spongiosus* grow well in rice and sawdust medium, while the former can also grow rapidly in sorghum and sawdust medium [12,13,62]. Adding 25% to 50% rice husk to eucalyptus sawdust can effectively increase the yield of *Pleurotus ostreatus* [63], suggesting that edible fungi varieties exhibit diverse utilization and requirements for cultivation substrates.

*B. xylophilus* has strong saprophytic properties. During the mycelial growth process, the cultivation substrates are gradually degraded, and the culture medium becomes soft. After casing with soil, one mushroom bag can mature 1–11 fruiting bodies, and the average fresh mushroom yield of *B. xylophilus* is 131.07 ± 29.38 g/bag, with a biological efficiency of 28.48 ± 6.39%. When *Buchwaldoboletus hemichrysus* or *B. lignicola* was inoculated on wood alone, the mass of the wood blocks decreased by 35~65% after 6 months of continuous culture [64], which indicated that the genus *Buchwaldoboletus* may have a strong ability to degrade lignin and cellulose.

## 5. Conclusions

In this study, the biological characteristics of carbon source, nitrogen source, inorganic salt, temperature, and pH in respect of the mycelial growth of *B. xylophilus* were identified. The artificial cultivation of *B. xylophilus* within a mushroom house was achieved for the first time, which is a new breakthrough in the cultivation of wild edible mushrooms and has great significance for the development of the edible mushroom industry. This technology presents numerous advantages, such as affordability, a brief cultivation cycle, substantial yields, and superior quality. Thereby, it is highly suitable for industrial-scale and extensive cultivation applications.

## Figures and Tables

**Table 1:** Effects of different carbon sources on the mycelial growth of *B. xylophilus*.

Carbon Source	Mycelial Growth Rate (mm/d)	Mycelial Growth Vigor
Dextrose	1.63 ± 0.07 ab	+++
Fructose	1.58 ± 0.06 ab	++++
Sucrose	0.64 ± 0.14 d	++
Mannitol	1.53 ± 0.09 b	+++
Maltose	1.70 ± 0.07 a	++++
Soluble starch	0.91 ± 0.09 c	+

Note: Different lowercase letters indicate a significant difference at *p* < 0.05. “+” means the mycelium was sparse and grew less, “++” means the mycelium was sparse and the growth was poor, “+++” means the mycelium was dense and thick, and “++++” means the mycelium was thick and robust, and grew well.

**Table 2:** Effects of different nitrogen sources on the mycelial growth of *B. xylophilus.*

Nitrogen Source	Mycelial Growth Rate (mm/d)	Mycelial Growth Vigor
Tryptone	1.46 ± 0.05 c	+++
Beef extract	1.41 ± 0.09 c	+++
Yeast extract	1.44 ± 0.09 c	+++
Ammonium tartrate	1.77 ± 0.04 a	++++
KNO_3_	1.27 ± 0.10 d	++
NH_4_NO_3_	1.58 ± 0.13 b	++++

Note: Different lowercase letters indicate a significant difference at *p* < 0.05. “++” means the mycelium was sparse and the growth was poor, “+++” means the mycelium was dense and thick, and “++++” means the mycelium was thick and robust, and grew well.

**Table 3:** Effect of different inorganic salts on the mycelial growth of *B. xylophilus.*

Inorganic Salt	Mycelial Growth Rate (mm/d)	Mycelial Growth Vigor
NaCl	1.42 ± 0.06 a	+++
MgSO_4_	1.49 ± 0.06 a	+++
KH_2_PO_4_	1.43 ± 0.13 a	+++
MnSO_4_	0.95 ± 0.09 c	++
CaSO_4_	1.42 ± 0.08 a	+++
NaNO_3_	1.27 ± 0.08 b	++

Note: Different lowercase letters indicate a significant difference at *p* < 0.05. “++” means the mycelium was sparse and the growth was poor, “+++” means the mycelium was dense and thick.

**Table 4:** Effect of temperature on mycelial growth.

Temperature (°C)	Mycelial Growth Rate (mm/d)	Mycelial Growth Vigor
15	0.00 ± 0.00 e	−
20	0.49 ± 0.08 d	+
23	0.99 ± 0.07 c	+
25	1.23 ± 0.04 b	+++
28	1.63 ± 0.10 a	++++
30	1.57 ± 0.12 a	+++
35	1.31 ± 0.06 b	+

Note: Different lowercase letters indicate a significant difference at *p* < 0.05. “−” means the mycelium has not germinated and grown, “+” means the mycelium was sparse and grew less, “+++” means the mycelium was dense and thick, and “++++” means the mycelium was thick and robust, and grew well.

**Table 5:** Effect of pH on mycelial growth.

pH	Mycelial Growth Rate (mm/d)	Mycelial Growth Vigor
4	1.12 ± 0.11 b	++
5	1.42 ± 0.14 a	+++
6	1.41 ± 0.07 a	+++
7	1.23 ± 0.09 b	++
8	0.84 ± 0.13 c	+

Note: Different lowercase letters indicate a significant difference at *p* < 0.05. “+” means the mycelium was sparse and grew less, “++” means the mycelium was sparse and the growth was poor, “+++” means the mycelium was dense and thick.

**Table 6:** Orthogonal experiment on mycelial growth of *B. xylophilus.*

Number	Carbon Source	Nitrogen Source	Inorganic Salt	Temperature	Mycelial Growth Rate (mm/d)	Mycelial Growth Vigor
1	1 (maltose)	1 (NH_4_NO_3_)	1 (MgSO_4_)	1 (25 °C)	1.02 ± 0.14 de	++
2	1 (maltose)	2 (ammonium tartrate)	2 (KH_2_PO_4_)	2 (28 °C)	1.76 ± 0.08 a	++++
3	1 (maltose)	3 (tryptone)	3 (CaSO_4_)	3 (30 °C)	1.29 ± 0.11 c	++
4	2 (dextrose)	1 (NH_4_NO_3_)	2 (KH_2_PO_4_)	3 (30 °C)	1.73 ± 0.11 a	+++
5	2 (dextrose)	2 (ammonium tartrate)	3 (CaSO_4_)	1 (25 °C)	1.13 ± 0.05 cd	++
6	2 (dextrose)	3 (tryptone)	1 (MgSO_4_)	2 (28 °C)	1.48 ± 0.19 b	+++
7	3 (fructose)	1 (NH_4_NO_3_)	3 (CaSO_4_)	2 (28 °C)	1.81 ± 0.07 a	++++
8	3 (fructose)	2 (ammonium tartrate)	1 (MgSO_4_)	3 (30 °C)	1.62 ± 0.12 ab	+++
9	3 (fructose)	3 (tryptone)	2 (KH_2_PO_4_)	1 (25 °C)	0.87 ± 0.13 e	++
K1	4.07	4.56	4.12	3.02		
K2	4.34	4.51	4.36	5.05		
K3	4.30	3.64	4.23	4.64		
X1	1.36	1.52	1.37	1.01		
X2	1.45	1.50	1.45	1.68		
X3	1.43	1.21	1.41	1.55		
R	0.09	0.31	0.08	0.67		

Note: Different lowercase letters indicate a significant difference at *p* < 0.05. Kn: Sum of mycelial growth rate at the nth level. Xn: The mean mycelial growth rate at the nth level. R: Range. “++” means the mycelium was sparse and the growth was poor, “+++” means the mycelium was dense and thick, and “++++” means the mycelium was thick and robust, and grew well.

**Table 7:** The average fresh mushroom yield and biological efficiency of *B. xylophilus*.

No.	Time of Primordial Formation (d) *	Primordial Number (Per Bag)	Number of Young Mushrooms (Per Bag) *	Number of Mature Fruiting Bodies (Per Bag) *	Fresh Mushroom Yield (g/bag) *	Biological Efficiency (%) *
1	11.28 ± 1.50	densely	5.70 ± 3.69	4.13 ± 2.21	131.07 ± 29.38	28.48 ± 6.39
	9~14		1~19	1~11	73.25~186.43	15.92~40.53

Note: “*” results are means ± SD and scope of 40 replicates.

**Table 8:** Sequences used in the analysis.

Taxon	Voucher	Locality	GenBank Accession Nos.		References
			28S	ITS	
*Buchwaldoboletus lignicola*	HKAS76674	Heilongjiang, China	KF112350	—	[6]
*Buchwaldoboletus lignicola*	HKAS84904	Germany	KT990538	—	
*Buchwaldoboletus lignicola*	KA14-0907	South Korea	—	MH170897	[18]
*Buchwaldoboletus lignicola*	3533	—	—	KM248950	Unpublished
*Buchwaldoboletus lignicola*	—	—	—	MH234512	Unpublished
*Buchwaldoboletus lignicola*	KM157323	—	—	GQ981493	[23]
*Buchwaldoboletus lignicola*	Pul1	Germany	JQ326997		[24]
*Buchwaldoboletus xylophilus*	FHMU5930	Yunnan, SW China	MW783417	MW783439	[19]
*Buchwaldoboletus xylophilus*	FHMU5930-1	Yunnan, SW China	MW783418	MW783440	
*Buchwaldoboletus xylophilus*	FHMU5931	Yunnan, SW China	MW783419	MW783441	
*Buchwaldoboletus xylophilus*	FHMU5931-1	Yunnan, SW China	MW783420	MW783442	
*Buchwaldoboletus xylophilus*	FHMU5932	Yunnan, SW China	MW783421	MW783443	
*Phlebopus portentosus*	N.K.Zeng22 (FHMU1)	Hainan, southern China	MW783433	MW783453	
*Phlebopus portentosus*	N.K.Zeng22-1 (FHMU1-1)	Hainan, southern China	MW783434	MW783454	
*Phlebopus portentosus*	N.K.Zeng1186 (FHMU753)	Hainan, southern China	MW783427	MW783447	
*Phlebopus portentosus*	N.K.Zeng5041-3 (FHMU5934-3)	Hainan, southern China	MW783438	MW783458	
*Phlebopus portentosus*	FHMU5935	Yunnan, SW China	MW783432	MW783452	
*Phlebopus portentosus*	FHMU5936	Yunnan, SW China	MW783435	MW783455	
*Phlebopus portentosus*	FHMU5937	Yunnan, SW China	MW783436	MW783456	
*Phlebopus portentosus*	FHMU5938	Yunnan, SW China	MW783437	MW783457	
*Buchwaldoboletus xylophilus*	FHMU5932-1	Yunnan, SW China	MW783422	MW783444	
*Buchwaldoboletus xylophilus*	—	India	—	ON707263	[22]
*Buchwaldoboletus xylophilus*	YITC-BU001	Yunnan, China (Artificial cultivation)	OR030332	OR029254	This study
*Buchwaldoboletus xylophilus*	YITC-BU002	Yunnan, China (Artificial cultivation)	OR030333	OR029255	This study
*Buchwaldoboletus xylophilus*	YITC-BU003	Yunnan, China (Artificial cultivation)	OR030334	OR029256	This study
*Chalciporus radiatus*	GDGM43285	Hunan, Central China	KP871800	KP871804	[25]
*Chalciporus radiatus*	GDGM50080	Hunan, Central China	KP871801	KP871806	
*Chalciporus radiatus*	GDGM43305	Guangdong, China	KP871802	KP871805	
*Chalciporus radiatus*	N.K.Zeng1379 (FHMU930)	Fujian, SE China	MH879710	MH885367	[26]
*Paxillus vernalis*	AFTOL-ID 715	—	AY645059	DQ647827	[27]
*Phlebopus mexicanus*	FCME:J.Cifuentes and S.Cappello-2009-233	Mexico	KM676001	KM675999	[28]
*Phlebopus mexicanus*	UJAT:S.Cappello-2507	—	KM676002	KM676000	
*Phlebopus portentosus*	REH8795	Thailand	FJ153623	EU718111	[29]
*Phlebopus roseus*	MY2017274	Sichuan, China	MK734306	MK734175	[30]
*Phlebopus roseus*	MY2017277_a	Sichuan, China	MK734305	MK734174	
*Phlebopus roseus*	MY2017275_a	Sichuan, China	MK734304	MK734173	
*Phlebopus roseus*	MY2017277	Sichuan, China	MK734303	MK734172	

## Data Availability

The sequence data generated in this study can be obtained from NCBI GenBank (http://www.ncbi.nlm.nih.gov/, accessed on 22 May 2023). The data included in this study are available through contacting the authors.
